# Attitudes and barriers to participation in window-of-opportunity trials reported by White and Asian/Asian British ethnicity patients who have undergone treatment for endometrial cancer

**DOI:** 10.1186/s13063-023-07572-x

**Published:** 2023-11-25

**Authors:** B. Mandane, A. Amirthanayagam, N. Patel, N. Darko, E. L. Moss

**Affiliations:** 1 Faculty of Health and Life Sciences, De Montfort University, Leicester, UK; 2 University Hospitals of Leicester NHS Trust, Infirmary Square, Leicester, LE1 5WW UK; 3 College of Life Sciences, University of Leicester, University Road, Leicester, LE1 7RH UK

**Keywords:** Endometrial cancer, Window-of-opportunity, Clinical trials, Ethnicity, Asian/Asian British

## Abstract

**Purpose:**

Window-of-opportunity trials (WOT) are a study design that have been used to investigate drug activity in endometrial cancer (EC). Recruitment to cancer clinical trials by patients from ethnic minority groups is reported to be lower than for patients of White ethnicity.

**Methods:**

A verbal questionnaire was conducted with White and Asian/Asian British ethnicity patients who had undergone treatment for EC. Strategic purposeful sampling was used to recruit patients from diverse social/educational backgrounds. Questions explored: background knowledge of clinical research, WOT study design, and views on medications that might be investigated. Thematic analysis was used to explore motivations for WOT participation and perceived barriers.

**Results:**

In total, 21 patients were recruited to the study (15 White and 6 Asian/Asian British). Views on optimum time to receive trial information differed, preferences ranging from 'at the time of diagnosis' to 'a few days after diagnosis'. The choice of medication under investigation had a strong influence on potential willingness to participate, with greater interest reported in medications derived from vitamins or food supplements rather than hormone-based drugs. Potential barriers to participation included concern over potential side-effects and the emotional/physical burden of a cancer diagnosis prior to major surgery.

**Discussion:**

This study provides important insights into patients’ views on WOT participation in EC and raises issues that need to be considered for future trial design and participant recruitment materials. The timing and format of study information and type of substance under investigation were factors influencing potential participation. Future studies should consider using multi-lingual visual information videos to address information needs, as this may encourage participation by ethnic minority patients.

**Supplementary Information:**

The online version contains supplementary material available at 10.1186/s13063-023-07572-x.

## Introduction

Window of Opportunity Trials (WOT) utilise the window-of-time (typically 2–3 weeks) before starting definitive treatment to investigate compounds on the target disease. In addition to advancing drug development, WOT can provide greater understanding of pharmacodynamics and mechanisms of action of the compound of interest, and can help to identify biomarkers, in order to optimise or personalise patient treatment selection [1]. WOT, however, can be associated with potential safety and logistical issues, including delaying standard first-line therapy in advanced/metastatic disease [2], patient risks-versus-benefits decisions and short time-frames.

Endometrial cancer (EC) is the most common gynaecological cancer in many countries with nearly 10,000 new cases diagnosed each year in the UK [3]. Although the long-term outcome for the majority of EC cases is very good, the efficacy of systemic therapies for metastatic/recurrent EC is low [4]. Studies have reported differences in the molecular profiles of ECs between women from different ethnic groups [5, 6], however clinical trials lack representation from these groups.

EC has become a focus for WOT due to the accessibility of the uterus for sample collection, with diagnostic biopsies typically being compared with tumour collected at the time of hysterectomy [7]. Compounds investigated to date have included repurposed drugs, such as metformin [8], and hormonal therapy, with the aromatase inhibitor anastrozole [9]. More recently, anti-cancer drugs such as the tyrosine kinase inhibitor dasatinib [10], the PARP inhibitor olaparib [11] and immunotherapy agents including nivolumab [12] and pembrolizumab (NCT03694834) are the focus of clinical trials. These studies have confirmed the utility of this study design to investigate a range of compounds, giving new insights into drug mechanisms and identifying tumour characteristics that could help personalise patient treatment.

Numerous factors have been shown to influence patient recruitment to WOTs, including perceived waiting times, prior research participation and higher education attainment [13]. The type of compound of interest has also been shown to impact on willingness to participate in studies aimed at chemoprevention. For example, willingness to participate was much higher if the study drug was a vitamin, food nutrient or increased the participant’s immunity, compared to if the study drug was a hormone-like or cytotoxic drug, of if the study had a placebo arm [13].

Recruitment of minority ethnic patients into clinical cancer research trials is relatively low. Data collected on participant recruitment at the University College London Hospitals NHS Foundation Trust found that recruitment levels into clinical trials were 30% lower for minority ethnic patients compared with White ethnicity cancer patients, after adjusting for disease, age and gender, and differed for each ethnic group [14]. Reasons for this will include system level barriers, individual barriers and interpersonal barriers [15]. Factors such as inclusion criteria may also play a role and therefore have to be considered carefully when designing a trial [16].

The city of Leicester is multi-ethnic with a high percentage of the population in the 2021 National Census identifying as Asian/Asian British ethnicity [17]. We have previously reported that the number cases of EC diagnosed within Leicestershire is rising in patients of Asian/Asian British ethnicity [18], and Asian ethnicity is the largest ethnic minority group of EC patients diagnosed in England [19]. It is therefore essential that clinical trials investigating EC treatments recruit participants that reflect the background EC population. In order to investigate perceived barriers to WOT participation in a multi-ethnic EC population, we conducted a study with patients who had undergone EC treatment within the previous 3 years in order to explore their experiences and attitudes towards information delivery, study design and agent of investigation.

## Methods

A verbal questionnaire study combining a series of structured questions with open questions and discussion was conducted between February 2019 and July 2021. Ethical approval for the study was granted by the North-West Liverpool East Research Ethics Committee (18/NW/0788).

### Participants

Patients who had undergone treatment for EC at the University Hospitals of Leicester NHS Trust within the previous 3 years and were clinically well with no sign of cancer recurrence were verbally invited to participate by a member of their clinical care team, and this was supported by a written invitation and information sheet. A strategy of purposeful sampling was used in order to recruit patients from diverse, social, ethnic and educational backgrounds. This was achieved through monitoring of the characteristics of recruited participants and focusing recruitment of participants with underrepresented characteristics. Patients’ self-designated ethnicity was categorised into two main categories White (W), which included individuals of British and Irish descent, and Asian/Asian British, which included individuals from specific UK Asian ethnic category groups such as Indian, Pakistani, and Bangladeshi. Throughout this manuscript, the term “Asian ethnicity” is used to refer collectively to individuals belonging to these Asian/Asian British groups. All patients provided written consent. Participants who did not speak English as their first language were offered an interpreter or family member to be present during the questionnaire.

### Data collection

The study consisted of a one-to-one verbal questionnaire with a member of the research team: BM (pharmacist) or EM (consultant gynaecological oncologist), both of whom had undergone training in qualitative research methods. BM had no prior relationship with the study participants; EM was a member of the clinical care team. Participants were informed of the interviewer characteristics in the study invitation letter. A series of structured questions (Supplementary data 1) were asked focusing on the participants’ pre-diagnosis knowledge of EC and clinical trials, and their experiences between diagnosis and definitive surgery. The structure and purpose of a WOT was explained by the researcher using a printed flow chart indicating timing of treatment and biopsies. The participant’s views on WOT recruitment, optimum timing and route of delivery of information were explored. Different classes of drugs that could be investigated were discussed and the participants' interest or concerns discussed. Answers provided to the questions were then explored using open questions following an interview schedule (Supplementary data 2), enabling investigation of the individuals’ rationale for their questionnaire responses and opinions regarding WOT participation. Potential solutions to proposed barriers to WOT participation were also discussed.

The questionnaire was developed and piloted amongst three clinicians working in gynaecological oncology and pharmacology to ensure readability prior to its implementation. The questionnaires were initially conducted face-to-face at the University Hospitals of Leicester; however, the COVID-19 pandemic resulted in a change to telephone questionnaires. Recruitment was continued until data saturation was reached, defined as when no new themes were generated. The interviews were audio recorded on an encrypted recorder and transcribed verbatim. Field notes were taken during the interviews and there were no repeat interviews.

### Data analysis

Participant responses and preferences from the structured questions were charted enabling quantitative analysis and descriptive statistics to be performed. Qualitative analysis of the participants’ responses to the open questions was performed using thematic framework analysis [20]. This was performed in three stages using an iterative approach: the coding of text ‘line-by-line’, the development of ‘descriptive themes’ from the original dataset and the generation of ‘analytical themes’. The latter analytical stage aimed to focus around the interpretation and generation of new explanations and hypotheses, building on the complexity and the richness of the dataset [21]. Open codes were assigned to the data, with explanatory notes. A second member of the research team reviewed four of the transcripts and the two reviewers compared codes and agreed on a set of codes to form the initial analytic framework. Coder reliability was conducted, and there was agreement and consistency between the independent coders. To ensure the researchers’ (EM, BM) preconceptions and biases did not influence decisions and actions throughout, reflexivity was also utilised as a strategy for improving rigour of the study. Specifically, to ensure that their preconceptions and biases did not influence their decisions and actions throughout the research process, the researchers engaged in prolonged engagement with the participants. This allowed them to gain familiarity and understanding of the participants’ experiences and context surrounding their participation in trials. By engaging in reflexivity, the researchers were able to determine how their own biases and preconceptions may have influenced the data collection and analysis process. They also sought to identify and address any potential bias by discussing their positioning and potential impact with wider members of the research team via regular group discussions. Reflexivity helped to ensure that the researchers were aware of their own biases and worked to minimise their impact on the research findings, as well as helping to increase the trustworthiness and credibility of the research results by demonstrating the researchers’ commitment to rigorous and unbiased research practices.

Further transcripts were reviewed, and the analytic framework applied, new codes were looked for and the analytic framework revised accordingly. This process was repeated until no new codes emerged. Coding software was not used. Once all transcripts had been revised, the final analytic framework was applied to all the transcripts and themes arranged using a thematic tree. The matrix was reviewed for connections between participants and categories. Transcripts were not returned to the participants for comment/correction.

## Results

In total, 21 women who identified with their sex assigned at birth participated in the study. Fifteen (71.4%) participants were of White ethnicity, and six were of Asian ethnicity (28.6%). Four of Asian participants reported that English was not their first language, and in one case, the questionnaire was completed with the assistance of an interpreter who was present with the researcher during the interview. The duration of the interviews was not recorded. A greater proportion of the White ethnicity participants lived alone, whereas a greater proportion of Asian participants did not drive a car (Table 1). The logistics of travelling to the hospital and work commitments were cited by both groups as the main barriers to visiting the hospital.

**Table 1:** Participant social/travel arrangements and perceived barriers to hospital attendance. Participants may have given more than one response

Participant social/travel arrangements and perceived barriers	White ethnicity (*n* = 15)	Asian/Asian British ethnicity (*n* = 6)
Lives alone		
Yes	5 (33.3%)	0
No	10 (66.7%)	6 (100%)
Drives a car		
Yes	13 (86.7%)	3 (50.0%)
No	2 (13.3%)	3 (50.0%)
Barriers to attending hospital appointment:		
Travel (parking, distance, public transport)	4 (26.7%)	2 (33.3%)
Work commitments	4 (26.7%)	3 (50.0%)
Family care responsibilities	3 (20.0%)	0
Financial cost	0	0
Needing support with transport	1 (6.7%)	1 (16.7%)
None	5 (33.3%)	3 (50.0%)

Eight of the White and four of the Asian ethnicity participants did not know of a family member, friend or colleague who had been diagnosed with EC. The internet and printed literature from healthcare professionals were the most common sources of information on health issues for White ethnicity participants, whereas information from family/friends/colleagues was reported by more Asian participants (Table 2). Previous clinical research study participation was reported by seven participants. Despite knowing about various different sources of information for clinical research studies, only eight participants reported having heard about clinical trials to develop new primary cancer prevention treatments.

**Table 2:** Knowledge and preferences of participants on methods of receiving health/research information. Participants may have given more than one response to each question

Participants' knowledge and preferences	White ethnicity (*n* = 15)	Asian/Asian British ethnicity (*n* = 6)
Sources of information to learn about health:		
Internet	9 (60.0%)	3 (50.0%)
Family/friends/colleagues	6 (40.0%)	4 (66.7%)
Books/journals	0	0
Patient support groups	1 (6.7%)	1 (16.7%)
Information leaflets from clinic	14 (93.3%)	4 (66.7%)
Previously participated in a research study (Yes)	5 (33.3%)	2 (33.3%)
Sources of information about research studies:		
Internet	1 (6.7%)	1 (16.7%)
Magazine/newspapers	3 (20.0%)	2 (33.3%)
Media (TV/radio/adverts)	7 (46.7%)	3 (50.0%)
Previous participation in a clinical trial	5 (33.3%)	1 (16.7%)
Friends/family/colleagues	9 (60.0%)	2 (33.3%)
Healthcare professionals	8 (53.3%)	2 (33.3%)
Heard of clinical trials to develop new treatments to prevent cancer (Yes)	5 (33.3%)	3 (50.0%)
Preference for receiving study information:		
At cancer diagnosis appointment	6 (40.0%)	3 (50.0%)
A few days after diagnosis	6 (40.0%)	0
At surgical pre-assessment clinic	1 (6.7%)	2 (33.3%)
Unsure	2 (13.3%)	1 (16.7%)
Method of receiving study information		
Information leaflet	1 (6.7%)	1 (16.7%)
Information leaflet/face-to-face discussion	12 (80.0%)	3 (50.0%)
Face-to-face/telephone call after a few days	5 (33.3%)	3 (50.0%)
Video	3 (20.0%)	5 (83.3%)
Email	1 (6.7%)	1 (16.7%)
Telephone call	1 (6.7%)	1 (16.7%)

There were mixed views on when recruitment to a WOT should be discussed with potential participants, with nine participants preferring at the time of cancer diagnosis, six a few days after diagnosis and three at the surgical pre-assessment clinic. Several participants highlighted that this decision would be dependent on the individual. A written information leaflet with a face-to-face discussion was the most favoured method of giving study information; however, Asian ethnicity participants preferred a link to a multi-lingual video explaining the study (Table 2).

Analysis of the participants comments identified five main themes influencing WOT participation.

- 1) Motivation for participation
  Altruism was a leading motivation for interest in WOT participation, with a willingness to advance research that brings advantages to others, even if it potentially meant putting themselves through a disadvantageous path: “Anything to help to prevent cancer in the future. I just think anything that is good, that helps other women to prevent them getting cancer.” (W3, 57 years). There was a greater consensus around the participants’ perceived understanding and willingness to advance medical knowledge: “Well if you don’t try it out, you are never going to know – are you? And if people won’t participate, you are not going to get anywhere, are you?”  (W2, 72 years). Factors that were suggested as potentially influencing participation included, stage at diagnosis, impact of a cancer diagnosis and a fear of delaying definitive treatment.
- 2) Barriers to participation
  Despite overall support for participation in research trials, many participants shared concerns regarding participation in a WOT and proposed potential barriers that could impact recruitment, including emotional/physical burden of a cancer diagnosis prior to major surgery, and perceived waiting time from diagnosis to curative surgery on the outcome. Practical arrangements were also highlighted, such as needing to take time away from work, extra appointments and travel to the hospital: “I think because you work full-time, time constraints can be difficult.”  (A1, 56 years). A higher number of Asian participants stated that travel arrangements were a barrier to their participation and that they needed support with transport (50%), as compared to White ethnicity participants (33.3%). This is not surprising given that 50% of Asian participants did not drive a car, compared to only 13.3% of White participants. Furthermore, pressures of work commitments were more apparent amongst Asian participants. However, the greatest concerns were centred around the ‘drug’ of interest and not wanting to make their state of ill health worse: “… well I’m already ill anyway and I wouldn’t want to put anything else in my body that could cause any more problems.” (W8, 57 years). One participant described strong feelings towards avoiding the use of “any medication” if possible, extending these emotions and comparing it to other values: “Even if I was getting paid thousand pounds, I would not want to take anything that I didn’t need to, unless you need to take it to keep yourself alive.” (W6, 59 years).
- 3) Drug under investigation
  Participants’ opinions and preferences varied greatly depending on the potential medicinal agent under investigation, although almost all reported that they would need detailed information before they made a decision to participate. When asked to give a preference to substances, ‘vitamin or food nutrient/supplement’ was the most popular option amongst both White and Asian participants, 66.7% for both groups. Known ‘anti-cancer drug’ or ‘existing licensed medication’ that was in use for another indication were less favoured with only four and two participants respectively from the whole study population preferring these options as their first choice of substance. ‘Hormone-like drugs’ were the least popular option, especially amongst White ethnicity participants. Only one participant listed ‘hormone-like drugs’ as their first choice and seven (85.7% White ethnicity) reported that this was the substance that they were least likely to choose. Examples of participants’ reasons for their opinions on the different classes of agent are contained in Table 3. A few participants questioned the impact of a drug over such a short-time interval and how the time factor impacted on their ranking preferences and choices of the investigational medicinal agent: “I think at the 2-weeks interval, the food and nutrients would be quite lower down because I think any of the food or nutrients – is … is a longer term/lifestyle thing.. just because I think its long-term lifestyle, hence the last choice.”  (W11, 70 years).
- 4) Timing of recruitment
  The timing of patient contact and the format of information resources inviting participation in a WOT was viewed as having a major influence on potential recruitment. It was acknowledged that potential participants may experience a great emotional impact from a cancer diagnosis and this would need to be considered when planning the optimum time to approach a potential participant: “At the time of diagnosis: for me personally, at the point of diagnosis. I wouldn’t want to wait.”  (W1, 73 years); whereas other participants had a different view: “So I don’t know how I would process this if you gave me this information at the same time as my diagnosis. (…).”  (A1, 56 years). The need for social support at the time of a cancer diagnosis and how this could impact on WOT participation was mentioned by one participant: “And it almost, isn’t that person or the cancer, it is the family around them. (…) My focus would have been on them, on my family. (…)” (A2, 51 years). The person delivering the study information and the importance of a good-doctor patient relationship were also felt to be influencing factors.
- 5) Information resources
  The need for an individualised approach to recruitment was highlighted. Many participants preferred a face-to-face discussion with none choosing printed/written resources alone. It was acknowledged that patients have different levels of knowledge of clinical research and education levels, and these could impact on comprehension of the study design and requirements, in particular if the information resources were only in written English. Videos on cancer management were acknowledged as a source of information “the family watched the operation on YouTube.”  (W5, 71 years), however, the need to provide printed resources and not just online resources was highlighted: “You’ve given stuff to take home, and I have read them. I haven’t got Internet.”  (W2, 72 years).

**Table 3:** Selected participant quotes on the potential different medications that could be used in a window of opportunity trial

Placebo *“If it is a dummy drug, I suppose it is the only way you can learn isn’t it. So I would go along with it, and hoping that I was the one getting the proper drug.” (W9, 54)*
Vitamins and nutrient supplements *“Yes, yes.. I feel safer and I believe that it would help. (…) I do look on the internet and you know these sorts of foods are great for cancer. Whether they are true or not, I am including those in my diet (…) So I very much believe in vitamins and nutrients.” (A1, 56)*
Drugs impacting the immune system *“I don’t think you can increase your immunity, I am not aware.” (A1, 56)*
Anti-cancer drug *“Hmm again, you are automatically drawn to that anticancer, I don’t know even then would definitely need more information. (…)” (W6, 59)*
Off-label drugs “*So, I think if it was licensed, I would assume that it would be safe to take. And I suppose I would probably take it, because more confidence – just because it has been tried and tested I suppose. (…)” (W3, 57)*
Hormone-like drugs *“(…) negative connotations that you hear from the press. I suppose it’s one of those things that’s linked to negative things when you hear it in the press in a negative sense.” (W4, 56)*

## Discussion

This study gives new insights into the views of patients who have recently undergone primary EC treatment and it identifies potential barriers and motivators that will be important to consider in future WOT design in order to maximise recruitment from ethnic minority populations. The results also raise other points that could impact on patient recruitment to WOT and oncology trials in general. Understanding the motivation for an individual to participate in a clinical trial and the influence of cultural background, beliefs and education is vital in guiding the future design of information resources. Although altruism is a commonly cited motivation [22], it is rarely the primary motivation for trial participation and other factors, such as potential side-effects, additional hospital visits and the views of friends or family are also reported to be influential [23, 24]. It is evident that access to transport and work commitments are influential to participation amongst Asian ethnicity participants. In light of this, further recognition should be given to patients/participants with intersectional identities and the practical challenges faced by potential participants. Greater barriers in access and recruitment to clinical trials can be experienced due to their employment, access to transport and ethnicity and this, along with appointment scheduling, needs to be considered in future trial design.

Given the time-critical nature of WOT recruitment, and the clinical importance of not delaying definitive surgery [25], the timing and format of information delivery needs to be considered. It is reported that 40–80% of the medical information provided by healthcare professionals is forgotten immediately and almost half of the information that is remembered is inaccurate [26]. Information on WOT participation will typically be given at/around the time of the patient’s cancer diagnosis, therefore devising strategies to avoid misinterpretation of information and resources that a potential participant can access with their family/friends in their own time may increase participation. The use of complex medical terminology, inter-individuality of the research team, the format of the information (e.g. discussion versus written) and factors relating to the patient (e.g. expectations, educational background) are all inter-dependent when trying to engage participants in research [26]. The role of multi-lingual information videos giving visual/verbal information about a clinical trial was suggested by several of the Asian participants as a better way of imparting information, rather through written information, since it was acknowledged that literacy levels may be lower in patients where English is not their first language. Future clinical trials should have multi-lingual written/audio/visual information resources readily available in a wide range of languages in order to support recruitment of participants from ethnic minority groups.

One of the biggest strengths of WOT is that they enable researchers to utilise the critical time period between diagnosis and surgical treatment to investigate the impact of a medicinal agent of the tumour. Their disadvantage, however, is often their short and finite treatment time frame, and clinically this could potentially result in insufficient time to observe significant pathological outcomes [8]. This point was identified by some participants and was suggested as a barrier to participation since it was felt that this may devalue the potential significance of the study in the participants’ view. Explanation of the role of potential surrogate end points, for example markers of tumour proliferation [27], may help overcome this potential barrier.

Findings from this study highlight how the choice of the investigational medicinal agent impacts on the participants’ willingness to take part in a trial. Vitamins or food supplements were the most chosen option by participants from both ethnic groups, with other classes of drugs being much less popular. Vitamins and dietary supplements have been the focus of many studies and many patients (20–80%) choose to take dietary supplements following a cancer diagnosis [28]. They are likely to be seen as the least ‘harmless’ and less likely to interact with existing medications. Our results also showed that ‘hormone-like drugs’ were the least favoured option. Media reports on the association of hormone replacement therapy (HRT) have had a negative influence on HRT usage [29] and may have influenced participants views in our study. This view is interesting, however, because progesterone-based therapies (levonorgestrel intrauterine device, megestrol acetate and medroxyprogesterone acetate) are standard treatment options for endometrial pre-cancer [30], and it is possible that a number of the study participants had received progesterone treatment previously. Clinician communication in explaining the potential side effects experienced with breast cancer adjuvant endocrine therapy was identified as being a factor in giving patients confidence to continue with treatment [31]. This highlights the need for adequate clinical support and additional sources of information with a WOT to answer participants’ queries regarding potential treatment effects, since this is likely in turn to impact participants’ compliance in taking the drug under investigation.

## Clinical implications

The findings of the study, as discussed above, have implications for trial recruitment and should be considered by clinicians when approaching patients, in order for trials to be representative of the population under investigation. There is onus on all clinicians to play their part in breaking down the barriers that inhibit research participation in diverse ethnic groups, in particular, implicit bias by the clinical team regarding their perceptions of patients’ willingness or suitability for recruitment needs to be addressed [32]. This can be achieved through strategies including, awareness training and education on the experiences of diverse populations [33], having a research team that reflects the diversity of the patient population [34] and utilising systems for overcoming practical barriers, for example multi-lingual information materials and interpreter support. Patient travel also should be considered, as was highlighted in our study, along with the location of the delivery of trials, utilisation of virtual technology to reduce in-person appointments and consideration of the timing of appointments, so as to minimise disruption on patients’ work commitments. The urgent need to address representation in gynaecological oncology clinical trials is being taken forward by the GOG Foundation and Society of Gynecologic Oncology with the IDEA initiative [35], including the proposal of establishing minimum thresholds for the inclusion of minority populations based on cancer incidence or mortality. This, along with other government strategies such as the NIH Minority Health and Health Disparities Strategic Plan 2021–2025, will help embed best practice within everyday trial recruitment. However, the real-world impact on participant recruitment of these developments will need to be prospectively monitored since mistrust is a commonly cited reason for non-participation by diverse race/ethnicity patients [36], and more fundamental changes in healthcare delivery will be needed to address wider health inequalities and inequity.

## Limitations

Although this study gives insights into WOT motivation, the views expressed cannot be taken as being representative of either the White or Asian populations resident in the UK due to the small number of participants. The Asian population resident within Leicester predominantly identify as Asian/Asian British: Indian (34.3%), whereas the representation from other Asian populations is lower: Pakistani (3.4%) and Bangladeshi (1.9%) [17]. It is therefore likely that our study participants were mainly from the Asian/Asian British: Indian population. Although Asian ethnicity is the most commonly reported minority group undergoing surgical treatment of EC in the UK [37] and the number of Asian patients diagnosed with EC is rising in Leicestershire [18], the potential number of Asian EC survivors available to participate in this study was lower than White ethnicity patients. The demographic details and reasons for declining recruitment were not collected, and this could have introduced bias with only participants who expressed interest in research agreeing to participate. Other limitations of this study are that the interview transcripts were not returned to the participants to check and that the questionnaire/interview duration was not recorded. Previous research participation is likely to be higher in our study population due to the high level of research activity within the Gynaecological Oncology department at the University Hospitals of Leicester.

## Conclusions

This study provides important insights into patients’ views on WOT participation in EC and raises issues that need to be considered for future trial design and participant information materials. The timing and format of the study information and the type of substance under investigation were factors influencing potential participation; however, the importance of practical issues including time away from work and transport to hospital appointments also needs to be considered. Asian ethnicity patients with intersectional identities often experience barriers that could adversely impact on participation in research studies such as WOTs. Recognition of intersectionality and an intersectional approach in future research will facilitate a more inclusive approach to WOT recruitment for Asian ethnicity patients who are undergoing treatment for EC. Future studies should consider practical arrangements and appointment schedules, as well as the availability of multi-lingual audio/visual information, for example videos, to address information needs as this may encourage participation by patients from ethnic minority populations.

### Supplementary Information

**Additional file 1:** **Supplementary data 1.** Study questionnaire.

**Additional file 2:** **Supplementary data 2.** Interview schedule.

## Data Availability

Data sharing is not applicable to this article due to ethical restrictions.
