# Supplementary material for: Attitudes and barriers to participation in window-of-opportunity trials reported by White and Asian/Asian British ethnicity patients who have undergone treatment for endometrial cancer
Source: Trials. 2023 Nov 25;24:754. doi: 10.1186/s13063-023-07572-x (PMC10676569; doi:10.1186/s13063-023-07572-x)
Supplement: Supplementary file 1 — Additional file 1: Supplementary data 1. Study questionnaire. [file 13063_2023_7572_MOESM1_ESM.pdf]

Supplementary data 1. Questionnaire

There are no right or wrong answers. We will ask questions in this meeting but **<u>you can skip any question that you do not wish to answer</u>**. This survey should take about 15 minutes to complete.

Part 1 – Background Information

1. How would you describe your ethnic background?

Ethnic Group	Tick
**(A) White**	
British	
Irish	
Any other white background (please write in line below)	
**(B) Mixed**	
White and Black Caribbean	
White and Black African	
White and Asian	
Any other mixed background (please write in line below)	
**(C) Asian or Asian British**	
Indian	
Pakistani	
Bangladeshi	
Any other Asian background (please write in line below)	
**(D) Black or Black British**	
Caribbean	
African	
Any other Black background (please write in line below)	
**(E) Chinese or other ethnic group**	
Chinese	
Any other (please write in line below)	
**(F) Other**	
**Do not wish to disclose.**	

2. Is English your first language?
   i. Yes
   ii. No, please state your first language ___________
3. What languages can you read and write fluently?
   a. English
   b. Gujarati
   c. Punjabi
   d. Tamil
   e. Urdu
   f. Hindi
   g. French

h. Spanish
i. Portuguese
j. Other, please state: ____

4. What is the highest level of education achievement you have completed?
   a. None
   b. Year 1-8
   c. Year 9-11
   d. Year 12 (high school diploma, or any school equivalent)
   e. 1-3 years of college (junior college)
   f. 4 years college (college degree)
   g. Masters
   h. Doctor, lawyer, (PhD, MD, JD)
   i. Other, please specify: _______________

5. Are you currently employed/self-employed?
   a. Yes, please state occupation: _____________
   b. No

6. Do you live alone?
   a. Yes
   b. No. If no, who lives with you? ____________

7. How would you describe your current relationship status?
   a. Married
   b. In a relationship. living with partner
   c. In a relationship, not living with partner
   d. Divorced, separated
   e. Widowed
   f. Single (never married)
   g. Other, please specify: _______________

8. Do you have children (any dependants?) Note: terminology guide: G[x]P[y].
   a. Yes

   b. No

9. If you would need to travel to the hospital – how would you do this? (Rating scale: 1 – 3 (most likely to least likely, respectively).
   a. By car
      i. Do you drive? Yes / No
      ii. If no, who drives you to the hospital? ______
   b. By bus
   c. By taxi
   d. Other, please specify: _______________

10. How reliable is your transportation to come to the hospital?
   a. Always reliable
   b. Somewhat reliable
   c. Sometimes unreliable
   d. Always unreliable

11. How long does it take you to travel to the clinic?

a. 0-1 hour
b. 1-2 hours
c. 2-4 hours
d. Don't know
e. Other: _____________________________

12. When you make arrangements to visit the hospital, what are the main barriers (limiting factors) to your decision-making?

a. Time off from work

b. Travel distance
c. Child care arrangements
d. Looking after / caring for family members
e. Financial costs
f. Anything else, please state: _______________________________

Part 2 – Endometrial (Womb) Cancer

13. Background knowledge: how much awareness do you have around endometrial cancer and its treatments?

a. No significant amount
b. Small amount
c. Average amount
d. Large amount

14. Have you actively sought out more information (about endometrial cancer) than that provided to you by your doctors, nurses and other healthcare professionals?

a. Yes (continue to question 15)
b. No (skip to question 16)

15. What sources of information do you normally use to learn more about your medical / health conditions? (select all that apply)

a. Internet
b. Family and friends
c. Books or journal articles
d. Cancer support groups
e. Brochures available in the clinic
f. Other, please specify: _______________

16. Do you know anyone with endometrial (womb) cancer?

a. Yes

If yes, who?

(i) Close family members
(ii) Distant family members
(iii) Friends
(iv) Work colleagues
(v) Other, please specify:___________________________________

b. No
c. Don't know

Part 3 – Clinical Trials

17. Have you heard of clinical research studies?

a. Yes
b. No
c. Examples (see Appendix A, guidance leaflet): ____

18. If yes, where have you heard about them?

a. Internet
b. Magazines / Newspapers
c. TV (television) / Radio / Public transport adverts
d. Have previously taken part in clinical trials
e. From family members
f. From hospital staff
g. Other: __________________________

19. Have you ever participated in any clinical research study?

a. Yes
b. No
   i. If no, please state reason why: _____________________________
c. Don't know

20. Have you heard about trials to develop new treatments to help **prevent** the development of cancer?

a. Yes
b. No

21. If your surgeon offered for you to participate in an endometrial cancer prevention research study prior to having your surgery, how would you feel about participating?

a. I would be willing to participate
b. I would NOT be willing to participate
c. I would be willing depending on the agent being tested
d. I would need more information to decide

22. If you were considering taking part in a study, **when** you would prefer to receive information about it? (Select all that apply, see Appendix B).

a. At the time of diagnosis

b. A few days after the diagnosis

c. At the surgical pre-assessment clinic

c. Other: ____

23. If you were considering taking part in a study, **how** would you prefer to receive information about it? (Select all that apply).

a. Information leaflet
b. Information leaflet with face-to-face discussion
c. Face to face meeting followed by a telephone call after a few days
d. Through a video link
e. Through email
f. Through a telephone call only
g. Other, please state: ____

24. Would you be happy for us to follow-up with a telephone chat a few days later?

A. Yes
B. No
C. Don't know
D. Other

25. In general, would you have been willing to participate in a study aimed to either develop new medicines or look into ways of using already existing medicines for alternative indications to **prevent** endometrial (womb) cancer? [Note: for patients with no EC if they had to undergo a hysterectomy would they be willing to participate?]

E. Yes
F. No
G. Don't know
H. I would need more information to decide
    a. Please share your thoughts: ___________________________________
    ____

26. On the other hand, if you were **diagnosed** with endometrial cancer, how likely would you then consider taking part in a research study considering finding new ways of treating it?

a. Very likely
b. Less likely
c. Not affected
d. Don't know
e. I would need more information to decide

Part 4 – Type of Substances

27. In general, how likely would you participate in an endometrial cancer **prevention** research study if you might receive *placebo (an inactive 'dummy' substance)* rather than the drug?

a. Very likely
b. Less likely
c. Not affected
d. Don't know
e. I would need more information to decide

28. In general, how likely would you participate in an endometrial cancer prevention (window trial) research study if the substance given was a *hormone-like drug*?

a. Very likely
b. Less likely
c. Not affected
d. Don't know
e. I would need more information to decide

29. In general, how likely would you participate in an endometrial cancer prevention research study if the substance given was a *vitamin or food nutrient / supplement*?

a. Very likely
b. Less likely
c. Not affected
d. Don't know
e. I would need more information to decide

30. In general, how likely would you participate in an endometrial cancer prevention research study if the substance given *increased your immunity (protection against infection)*?

a. Very likely
b. Less likely
c. Not affected
d. Don't know
e. I would need more information to decide

31. In general, how likely would you participate in an endometrial cancer prevention research study if the substance was *given in combination with a proven anticancer drug*?

a. Very likely
b. Less likely
c. Not affected
d. Don't know
e. I would need more information to decide

32. In general, how likely would you participate in an endometrial cancer prevention research study if the substance *given was already a widely-used licensed medicine but for another indication (e.g. anti-diabetic medicine being tested for an alternative indication)*?

a. Very likely
b. Less likely
c. Not affected
d. Don't know
e. I would need more information to decide

33. Any other comments, on which type of substances you would be more willing to take as part of the clinical trial (examples: food supplements, nutrients, hormone-like medicines, existing licensed medicines but used for another indication, etc).

____

Part 5 – Conclusions

35. Is there anything else you would like to add?

____
