# Supplementary material for: Attitudes and barriers to participation in window-of-opportunity trials reported by White and Asian/Asian British ethnicity patients who have undergone treatment for endometrial cancer
Source: Trials. 2023 Nov 25;24:754. doi: 10.1186/s13063-023-07572-x (PMC10676569; doi:10.1186/s13063-023-07572-x)
Supplement: Supplementary file 2 — Additional file 2: Supplementary data 2. Interview schedule. [file 13063_2023_7572_MOESM2_ESM.pdf]

Supplementary data 2: Study interview schedule

Introductions.

Background to the project and explanation of project aims.

Complete consent form and answer any questions, ensure that participants have read and understood the participant information sheet.

Housekeeping.

Patient experiences

Explore language preferences / educational experience where appropriate;

Background knowledge about clinical trials (e.g. research awareness, how the patient heard of it – e.g. internet, friends, newsletters, charities, etc);

Ever taken part in clinical research studies? If so, what was their experience of it?

In general, would the patient have been willing to participate in a study aimed to either develop new medicines or look into ways of using already existing medicines for alternative indications to prevent endometrial (womb) cancer?

If the patient was considering taking part in a study, how would he/she prefer to receive information about it? (prompts: information leaflet; information leaflet with face-to-face discussion; face-to-face meeting followed by a telephone call after a few days; through a video link; through email; through a telephone call only; other).

Explain what a window trial is

Is this the sort of trial the patient might consider to take part in?

Prompt – What would be their main motivation?

Which type of substances would the patient be more willing to take as part of the clinical trial (examples: food supplements, nutrients, hormone-like medicines, existing licensed medicines but used for another indication, substances with proven anticancer properties for another indication, substances to increase immunity, etc).

- *Thoughts on receiving placebo (an inactive 'dummy' substance)?*

Screening for womb cancer

Explore the patient's background knowledge on endometrial (womb) cancer (prompts: anyone in the family or friends' circle affected? What information sources does the patient use to learn more about it?)

Barriers to study participation: explore themes around family opinions, childcare commitments, hospital travel appointments; travel time plus time commitment to the research; employment – difficulty in getting time off from work; financial costs; carer arrangements;

Thoughts: what would encourage the patient to take part in such a study? (try to explore language and cultural barriers here)
